# Defining genetic and chemical diversity in wheat grain by 1H‐NMR spectroscopy of polar metabolites

**DOI:** 10.1002/mnfr.201600807

**Published:** 2017-02-24

**Authors:** Peter R. Shewry, Delia I. Corol, Huw D. Jones, Michael H. Beale, Jane L. Ward

**Affiliations:** ^1^ Department of Plant and Biology and Crop Science Rothamsted Research West Common, Harpenden Hertfordshire UK

**Keywords:** Grain Composition, Metabolomics, NMR Spectroscopy, Transgenic, Wheat

## Abstract

**Scope:**

The application of high‐throughput 1H nuclear magnetic resonance (1H‐NMR) of unpurified extracts to determine genetic diversity and the contents of polar components in grain of wheat.

**Methods and results:**

Milled whole wheat grain was extracted with 80:20 D_2_O:CD_3_OD containing 0.05% d_4_–trimethylsilylpropionate. 1H‐NMR spectra were acquired under automation at 300°K using an Avance Spectrometer operating at 600.0528 MHz. Regions for individual metabolites were identified by comparison to a library of known standards run under identical conditions. The individual 1H‐NMR peaks or levels of known metabolites were then compared by Principal Component Analysis using SIMCA‐P software.

**Conclusions:**

High‐throughput 1H‐NMR is an excellent tool to compare the extent of genetic diversity within and between wheat species, and to quantify specific components (including glycine betaine, choline, and asparagine) in individual genotypes. It can also be used to monitor changes in composition related to environmental factors and to support comparisons of the substantial equivalence of transgenic lines.

**Abbreviations**
Eβf: E‐β‐farnesene
1H‐NMR: 1H nuclear magnetic resonance
PCA: Principal Component Analysis
TSP: d_4_–trimethylsilylpropionate

## 1 Introduction

Wheat is the third most important cereal crop in the world in terms of total production, the annual production over the 5‐year period from 2008 to 2012 averaging about 680 million tonnes (http://faostat.fao.org/site/339/default.aspx). However, it is the most widely grown cereal, being the dominant crop in temperate zones and grown from Scandinavia to the south of Argentina, including highlands in the tropics 1. The demand for wheat‐based foods is also increasing in countries undergoing urbanization and industrialization, including countries which are outside its area of adaptation (such as parts of sub‐Saharan Africa) 2. Although the contribution of wheat to total calorific intake is about 20% in Western Europe, it can range between 50 and 70% in some countries in North Africa and in West and Central Asia.

Cultivated wheat comprises diploid, tetraploid, and hexaploid species. These species have one, two, and three genomes, respectively, each genome comprising seven pairs of chromosomes. The cultivation of wheat probably started about 10 000 years ago as part of the Neolithic Revolution which saw a transition from hunting and gathering to settled farming and the cultivation of crops. The earliest cultivated forms of wheat were diploid einkorn (*Triticum monococcum* var. *monococcum*, genome AA) and tetraploid emmer (*T. turgidum* var. *dicoccum*, genomes AABB) which probably originated from wild grasses in the south‐eastern part of Turkey 3. Cultivated bread wheat, which is hexaploid (genomes AABBDD), probably first appeared in the Middle East about 9000 years ago 4 and has since migrated across the temperate world. Most of the wheat grown globally is bread wheat (*T. aestivum*), but other species are grown to a lesser extent, notably about 35–40 million tonnes a year of durum (also called pasta) wheat (*T. turgidum* var. *durum*), a tetraploid species related to emmer, which is grown in the hot dry climate of the Mediterranean and similar areas. In addition, small volumes of “ancient” wheat species are grown for traditional foods or to satisfy the increasing demand for “healthy” alternatives to modern bread and pasta wheats. These are einkorn, emmer (which are discussed above), and spelt which is a hexaploid form related to bread wheat (*T. aestivum* var. *spelta*). These ancient wheat species are “hulled,” in that the glumes adhere tightly to the grain and need to be removed before processing, whereas modern bread and durum wheats are free threshing.

The spread of wheat has been associated with the development of a wide range of diversity which provides adaptation to different environments. This diversification is facilitated by high genome plasticity 3 and has resulted in many thousands of distinct genotypes and cultivars (estimated as over 25 000 by 1).

This diversity is exploited by breeders to improve the yield, agronomic performance, and end use quality, notably for processing into bread, other baked goods, pasta, and noodles. However, in recent years it has also become of interest in relation to increasing the contents and improving the compositions of components that contribute to health, in particular dietary fiber components and “bioactive” phytochemicals (notably terpenoids and phenolics). However, these components require expensive and time‐consuming analyses, and are difficult to determine in rapid high‐throughput screening procedures.

Metabolomic profiling is a well‐established tool to study diversity in plant composition, including cereals 5, with a range of platforms based on mass spectroscopy 6, 7 or NMR spectroscopy. We have developed high‐throughput 1H nuclear magnetic resonance (1H‐NMR) analysis of unpurified extracts made directly into deuterated aqueous methanol as a routine screening tool in plant metabolomics 8, 9. This method can be applied without modification to a range of plant tissues, including wheat grain 10, with data analysis carried out using commercially available software. We therefore illustrate the use of this method to determine the extent of diversity in grain composition of bread wheat and other wheat species, in relation to defining genetic diversity, quantifying components of relevance to diet and health and exploring unintended effects on the composition of grain in transgenic wheat.

## 2 Materials and methods

### 2.1 The HEALTHGRAIN wheat samples

Wholemeal samples from the EU HEALTHGRAIN project were as described in 11, 12. These comprised 150 bread wheat genotypes, 130 winter type and 20 spring type, selected to represent the range of diversity available to breeders. They therefore have wide geographical diversity in origin (from Europe to East Asia, the Americas, and Australia) and include old varieties and landraces, breeding lines and modern cultivars. In addition, five modern cultivars of spelt, ten of durum wheat, and five each of two early cultivated forms of wheat, einkorn, and emmer were also included. Finally, ten lines of rye (*Secale cereale*), five of oats (*Avena sativa*), and ten of barley (*Hordeum vulgare*) were selected, using similar criteria to those used to select the wheats. All lines were grown together in single plots in Martonvasar, Hungary, in 2004–2005.

Twenty‐three lines (21 winter and three spring type) were selected from the 150 and grown with three additional spring lines in single plots on the same site in 2005–2006 and 2006–2007, and on sites in the United Kingdom (Saxham) and France (Clermont‐Ferrand) in 2006–2007. The 24 winter lines only were also grown on a further site in Poland (Choryn) in 2006–2007.

### 2.2 The transgenic wheat samples

The UK wheat cultivar Cadenza was transformed with DNA constructs designed to constitutively express a plastidially targeted form of the enzyme E‐β‐farnesene (Eβf) synthase, either alone, or in combination with another cassette designed to constitutively express a plastidially targeted form of farnesyl diphosphate synthase, the precursor of Eβf in the pathway. Many of the 81 GM wheat events obtained were found to emit Eβf and two (labeled as GM1 and GM2) were chosen for further analysis and field‐trialing. GM2 (B2812 R9P1) possessed only the gene encoding Eβf synthase (with four copies of the gene per haploid genome) and displayed Eβf emission in the mid‐range of our observations. GM1 (B2803 R6P1) possessed one copy of the EβfS gene plus one copy of the farnesyl diphosphate synthase gene per haploid genome and displayed a high level of Eβf emission. The transgene insertions were stably inherited via simple 3:1 Mendelian ratios in the chromosomal DNA and the plants used in the field trial were all homozygous. All the plants that emitted Eβf had an otherwise normal phenotype, were fully fertile, and did not display any obvious evidence of somaclonal mutations 13. These GM wheat plants were grown in 2012–2013 in experimental field trials at the Rothamsted Research farm using a 4 × 4 Latin square design with randomized 6 m × 6 m plots (four GM1, four GM2, and eight plots of untransformed cv Cadenza controls). Seeds were harvested in September 2013, stored in dry conditions at 20°C and 1H‐NMR analysis performed on milled whole grain in 2014.

### 2.3 1H‐NMR spectroscopy

1H‐NMR sample preparation was carried out according to the procedures described in 5, 7. Wholemeal samples (30 mg) were extracted in triplicate using 80:20 D_2_O:CD_3_OD containing 0.05% d_4_–trimethylsilylpropionate (TSP; 1 mL) as internal standard. Samples were extracted at 50°C for 10 min. After centrifugation (5 min at 13 000 rpm), the supernatant was removed and heated to 90°C for 2 min to halt enzyme activity. After cooling and further centrifugation, the supernatant (650 μL) was transferred to a 5 mm NMR tube for analysis.

1H‐NMR spectra were acquired under automation at 300°K using an Avance Spectrometer (BrukerBiospin, Coventry, UK) operating at 600.0528 MHz and equipped with a 5 mm selective inverse probe. Spectra were collected using a water suppression pulse sequence with a 90° pulse and a relaxation delay of 5 s. Each spectrum was acquired using 128 scans of 64 000 data points with a spectral width of 7309.99 Hz. Spectra were automatically Fourier‐transformed using an exponential window with a line broadening value of 0.5 Hz. Phasing and baseline correction were carried out within the instrument software. 1H chemical shifts were referenced to d_4_–TSP at δ0.00.

1H‐NMR spectra were automatically reduced, using Analysis of MIXtures software (BrukerBiospin), to American Standard Code for Information Interchange files containing integrated regions or “buckets” of equal width (0.001 ppm for quantitation of asparagine and 0.01 ppm for multivariate analysis (1005 binned regions)). Spectral intensities were scaled to the d_4_–TSP region (δ0.05 to –0.05). The American Standard Code for Information Interchange file was imported into Microsoft Excel for the addition of sampling/treatment details. Signal intensities for characteristic spectral regions for 25 major metabolites (with baseline resolved peaks) were extracted via comparison to library spectra of known standards run under identical conditions. Quantitation against a known concentration of d4–TSP was carried out using the known number of hydrogens responsible for each characteristic peak of each metabolite.

### 2.4 Statistical analysis

Principal Component Analysis (PCA) was conducted on quantified data scaled to unit variance using SIMCA‐P software (version 13, MKS Umetrics). Quantified data from triplicate extraction replicates were averaged prior to multivariate analysis. PCA from 1H‐NMR fingerprint data was carried out using mean‐centered scaling.

## 3 Results and discussion

### 3.1 Diversity of composition in bread wheat

**Figure 1:**
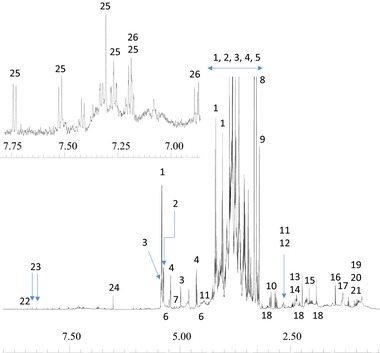
Typical 600 MHz 1H‐NMR spectrum of wholemeal flour extracted with 20:80 CD_3_OD:D_2_O. 1: sucrose; 2: maltose; 3: raffinose; 4: glucose; 5: fructose; 6: galactose; 7: galactinol; 8: glycine betaine; 9: choline; 10: asparagine; 11: malate; 12: citrate; 13: glutamine; 14: glutamate; 15: acetate; 16: alanine; 17: threonine; 18: GABA; 19: valine; 20: leucine; 21: isoleucine; 22: formate; 23: adenosine; 24: fumarate; 25: tryptophan; 26: tyrosine.

Figure 1 shows a typical 1H‐NMR spectrum of a polar extract of wholemeal wheat flour. The spectrum can be broadly divided into three regions. The central part, about δ3 to δ4.3 comprises overlapping peaks corresponding to abundant carbohydrates. These peaks are flanked by regions corresponding to anomeric protons of sugars and the aliphatic region of the spectrum which includes signals arising from organic acids, aliphatic amino acids and other polar low molecular mass components (notably choline and glycine betaine). The aromatic region includes signals relating to aromatic amino acids such as tryptophan. A comparison was therefore carried out using polar extracts of wholemeal flours of 150 wheat lines grown on one site as part of the EU HEALTHGRAIN project 11. These lines were selected to represent the range of diversity in the gene pool available for wheat breeders.

**Figure 2:**
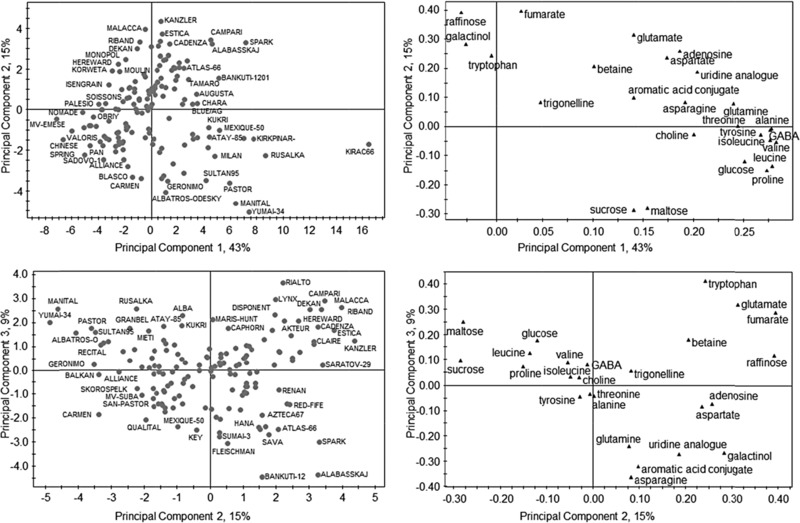
PCA analysis of quantified major metabolites from 1H‐NMR (600 MHz) analysis of CD_3_OD:D_2_O (1:4) extracts of wholemeal flour of 150 bread wheat lines grown at a single location in 2004–2005.

The levels of known metabolites with characteristic, baseline resolved peaks were quantified directly from the spectral data based on comparison with a library of reference spectra run under the same acquisition conditions, and the resulting table used for onward statistical analysis. Hence, the multivariate analysis shown in Fig. 2 not only allows us to compare the diversity of the genotypes (PCA scores plot, left panels) but also to identify the major metabolites which are responsible for the separation (PCA loadings, right panels). Thus, the top panels of Fig. 2 show that the genotypes can be separated on the basis of their polar metabolome with PC1 accounting for 43% of the variation and PC2 accounting for 15% of the variation within the dataset. Genotypes residing on the right hand side (e.g. Kirac66) of the PCA scores plot contained elevated levels of aliphatic amino acids and elevated glucose. Mv‐Emese, residing on the left hand side of the scores plot, contained less of these components but elevated levels of raffinose, galactinol and tryptophan. PC2 described the separation of genotypes in the vertical direction and genotypes residing in the lower half of the PCA scores plot, such as Yumai 34 and Manital contained higher levels of disaccharides (sucrose and maltose). This was in contrast to genotypes residing at the top of the PCA scores plot (e.g. Kanzler and Malacca) which contained lower disaccharide levels but higher than average levels of raffinose, fumaric acid, and glutamic acid. The lower panels of Fig. 2 include the PCA scores and loadings for PC2 (15%) versus PC3 (9%). Genotypes with a low score in PC3 (e.g. Bankuit‐12 and Alabasskaj) reside at the bottom of the PCA scores plot and analysis of the corresponding loadings plot indicate that these samples are lower in asparagine, glutamine and galactinol.

It has been suggested that intensive plant breeding, including wheat breeding, has led to a reduction in diversity in modern cultivars, increasing their susceptibility to pests and pathogens and limiting progress in breeding for novel targets such as increasing the contents of health‐promoting components. Figure 2 therefore provides a measure of “in species” diversity which can be used, together with molecular markers, to select appropriate lines to increase diversity in wheat breeding.

### 3.2 Comparison of diversity between wheat species

It is also of interest to compare the diversity in bread wheat to that in other wheat species, particularly “ancient wheats” which are often suggested to have health benefits compared to modern bread wheat (although the evidence for this is inconclusive, see 14).

**Figure 3:**
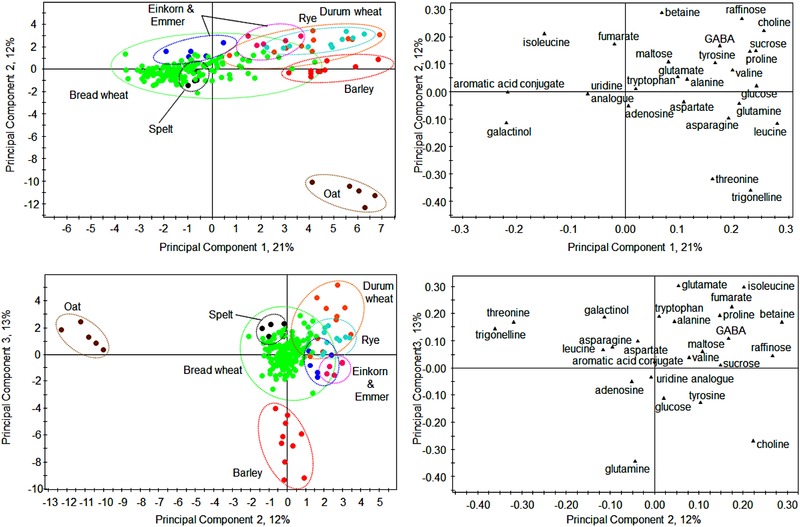
PCA of quantified major metabolites from 1H‐NMR analysis of CD_3_OD:D_2_O (1:4) extracts of wholemeal flour of 150 bread wheat lines and 50 other cereal lines grown at a single location in 2004–2005.

The EU HEALTHGRAIN study also included small numbers of genotypes of other wheat species, ten durum wheat, five spelt, five einkorn and five emmer, as well as ten barley, ten rye, and five oat genotypes. Comparison of the quantified metabolite profiles of these species (Fig. 3) therefore provides information on the diversity within and between these species and bread wheat, although it must be stressed that the sample numbers are small. Nevertheless, it is clear that the ancient wheat species fall largely within the same diversity limits as bread wheat, whereas rye and durum wheat show overlaps and barley and oats clearer separations. Analysis of the PCA scores and loadings plots for PC1 and PC2 showed a clear separation of the oat samples which was due to higher levels of threonine and trigonelline compared to samples from other species. When PC3, accounting for 13% of the variance, was analyzed against PC2 the PCA scores plot showed a clear separation of the barley samples. Analysis of the corresponding loadings plot indicated that this was due to increased glutamine in these samples. Metabolite profiling therefore allows the limits of diversity both within and between cereal species to be defined.

### 3.3 Determination of bioactive components by 1H‐NMR screening

The datasets used in Fig. 2 have also been used to extract data on bioactive components which may have impacts on diet and health. These are the methyl donors choline and glycine betaine and the amino acid asparagine.

In humans, methyl donors contribute to the reduction of plasma homocysteine. Hyperhomocysteinemia is a major risk factor in cardiovascular disease, with the homocysteine produced by demethylation of methionine being removed either by remethylation to methionine, metabolism to give cysteine or conversion to *S*‐adenosylhomocysteine. The remethylation of homocysteine requires a methyl donor, either folate (vitamin B9) or glycine betaine ((*N*,*N*,*N*,‐trimethyl) glycine) or choline. Glycine betaine and choline can also substitute for folate in other methylation reactions including the methylation of DNA 15, 16, 17. Humans obtain glycine betaine almost solely from their diet, but it can also be produced by the irreversible conversion of choline. Wheat contains the highest reported levels of glycine betaine of all plant foods, 12.9 and 15 mg/g in bran and 2.91 mg/g in whole grain, with lower levels of free choline (about 0.5 mg/g in bran and 0.14 mg/g in whole grain) 18, 19. Furthermore, dietary intervention studies have shown that the concentration of betaine is increased in the serum after the consumption of whole grain or bran‐rich cereal products 20, 21 and that betaine is converted in mammals to amino acid‐derived betaines which could have additional metabolic roles 22. Analysis of the 1H‐NMR dataset used in Fig. 2 showed that the content of glycine betaine in wholemeal flour of the 150 HEALTHGRAIN wheat lines varied from 0.97 to 2.94 mg/g and of choline from 0.18 to 0.28 mg/g dry weight 23.

Asparagine is of interest as a precursor of acrylamide which is formed in processed foods by a Maillard reaction with reducing sugars 24, 25 and may be present in cooked foods at concentrations up to 1 mg/kg 26, 27. The formation of acrylamide is correlated with the free asparagine content of wheat flour, rather than the content of reducing sugars 28, 29. In the dataset shown in Fig. 2, the concentration of asparagine in the 150 wheat lines varied from 0.32 to 1.56 mg/g dry weight 30.

These two components (glycine betaine and asparagine) also provide a good illustration of an important concern affecting the interpretation of plant metabolomics, which is the extent to which the differences observed are determined by genetic differences between the lines (G), the effects of environment (E), or interactions between these two factors (G × E). In the datasets presented in Figs. 2 and 3, environmental impacts are minimized by ensuring that the samples are grown together under the same conditions and the same farm management. However, this will not eliminate specific G × E interactions originating from, e.g. differences in dates of heading, flowering and grain maturation between the species.

**Figure 4:**
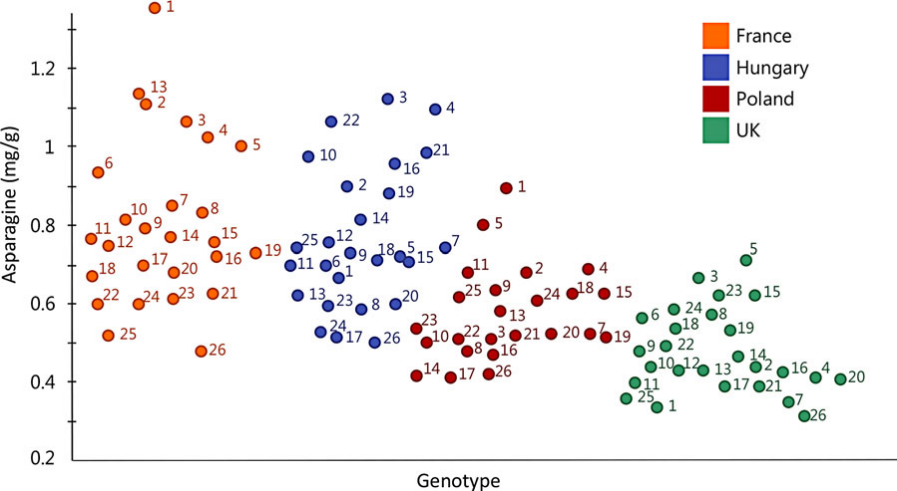
Asparagine concentrations (mg/g), determined by 1H‐NMR, of wholemeal flour of 26 bread wheat genotypes grown at four locations in 2006–2007 within the EU HEALTHGRAIN project. Data points are colored according to site (Hungary: blue; France: orange; UK: green; Poland: red (24 lines only)) and numbered according to the individual genotypes. 1: Estica; 2: Disponent; 3: Lynx; 4: Riband; 5: San‐Pastore; 6: Cadenza; 7: Tommi; 8: Maris‐Huntsman; 9: CF99105; 10: Campari; 11: Avalon; 12: Chinese‐Spring; 13: Crousty; 14: Herzog; 15: Spartanka; 16: Malacca; 17: Isengrain; 18: Obriy; 19: Tremie; 20: Tiger; 21: Rialto; 22: Claire; 23: Mv‐Emese; 24: Gloria; 25: Atlas‐66; 26: Valoris.

Analysis of sets of genotypes grown in multiple environments allows the contributions of G, E, and G × E to be estimated, with G providing an estimate of the “broad sense heritability” of the trait. In the EU HEALTHGRAIN study, the analysis of 23–26 genotypes grown in either four or six environments allowed the heritability of a range of components to be calculated 12. The heritabilities calculated for the components discussed here are particularly low: 0.36 for glycine betaine, 0.25 for choline 23, and 0.13 for asparagine 30. The impact of the environment is illustrated in Fig. 4, which shows the contents of asparagine (determined from the NMR spectra) in wholemeal flour of 26 genotypes (numbered 1–26) grown on field sites in four countries in 2006–2007.

### 3.4 Determination of unintended effects of transgenesis on wheat grain composition

Transgenic wheat has been available for over 20 years but, by contrast with maize, soybean and a number of other major crops, has not been developed for commercial production (reviewed by 31). Most of the reasons for this lack of development are outside the scope of this article, but one concern which is often raised is whether the process of transgenesis can have unintended consequences on grain composition which may have impacts on food safety and quality for diet and health. Consequently, establishing the “substantial equivalence” of transgenic crops to their non‐GM comparator is of key interest to breeders, regulators, and consumers.

The 1H‐NMR spectroscopy approach described here was initially developed and evaluated for a study of the substantial equivalence of transgenic wheat which had been engineered to have increased dough strength by expression of high levels of high molecular weight subunits of glutenin in the developing grain 32. Transgenic and control lines were grown in replicate field trials at two sites in the United Kingdom for 3 years (harvested 1998–1999, 1999–2000, 2000–2001) and their grain composition and quality compared 33. Multivariate analysis of the 1H‐NMR fingerprint data of polar extracts of white flour showed stronger effects of sites and years than of genotype, although some differences were observed between one line showing very high transgene expression and its corresponding untransformed control line 10. However, effects on grain composition were to be expected in this material as substantial and intended changes in grain protein content and composition were achieved.

More recently we have used the same approach to compare field grown grain of a second type of transgenic wheat. This was engineered to express the genes necessary to generate the terpene Eβf, an aphid alarm pheromone, to drive aphid pests away from the crop 13.

**Figure 5:**
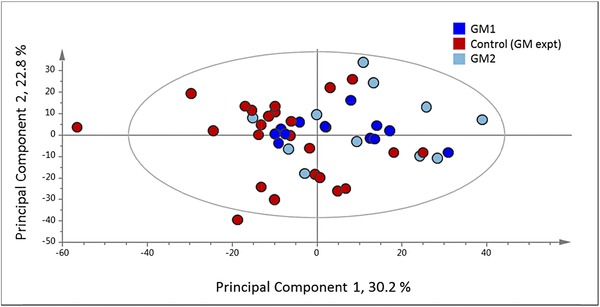
PCA of whole fingerprint data from 1H‐NMR analysis of CD_3_OD:D_2_O (1:4) extracts of wholemeal flour of two transgenic lines and one control line of wheat cv Cadenza grown in the field in 2013. Four replicate plots of each transgenic line and eight replicate plots of the control line were analyzed each with three technical replicates.

**Figure 6:**
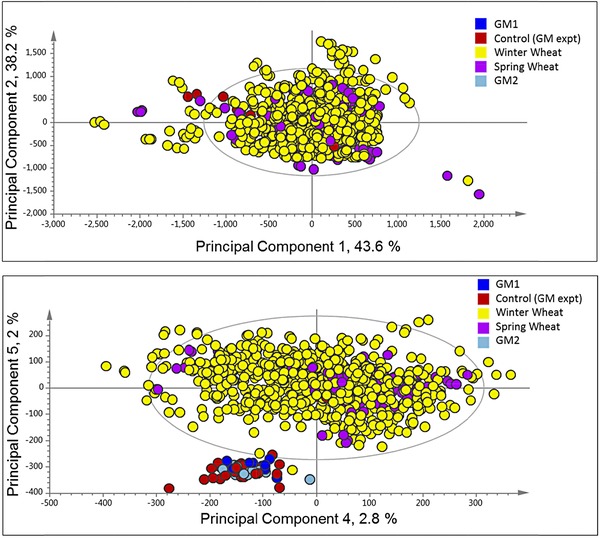
PCA of whole fingerprint data from 1H‐NMR analysis of CD_3_OD:D_2_O (1:4) extracts of wholemeal flour of two transgenic lines and one control line of wheat cv Cadenza grown in the field in in the United Kingdom in 2013 (data as used in Fig. 5) combined with analyses of 130 winter and 20 spring wheat lines grown in the field in Hungary in 2007 (data as used in Fig. 2).

In order to utilize the full information present in the spectra, the individual 1H‐NMR peaks were treated as biochemical signatures, and used for multivariate analysis without prior annotation of individual metabolites. The 1H‐NMR spectra of polar extracts from milled whole grain of two GM and one control line were initially compared by PCA (Fig. 5), which showed incomplete separation of the two transgenic lines and the control but no separation between the transgenic lines themselves. In order to determine the relevance of this separation in the context of the wider diversity in wheat grain composition, the dataset from the GM trial (obtained in 2014) was combined with the HEALTHGRAIN dataset used in Fig. 2 (obtained in 2010; Fig. 6). The scores plot of PC1 (44% of the variation) versus PC2 (38%) showed no separation of the transgenic or control lines from the HEALTHGRAIN lines (Fig. 6). However, when PC4 and PC5 (which together account for only 4.8% of the total variation) are plotted (Fig. 6), a clear separation is observed between the HEALTHGRAIN genotypes and the transgenic experiment, but not between the transgenic and control lines in the latter. It is therefore likely that the separation resulted from the environmental differences between the sites in Hungary in 2004–2005 (HEALTHGRAIN) and the United Kingdom in 2012–2013 (transgenic experiment).

**Figure 7:**
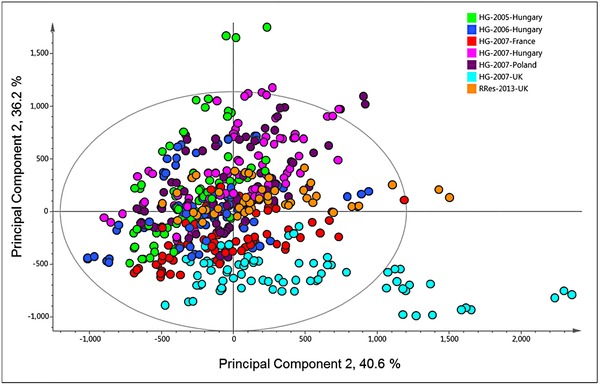
PCA of whole fingerprint data from 1H‐NMR analysis of CD_3_OD:D_2_O (1:4) extracts of wholemeal flour of two transgenic lines and one control line of wheat cv Cadenza grown in the field in the United Kingdom in 2012–2013 (data as used in Fig. 5; shown in orange as RRes 2013) combined with analyses of wheat lines grown in the field in Hungary in 2004–2005 (23 lines) and 2005–2006 (26 lines) and in Poland (24 lines), Hungary, France, and the United Kingdom (26 lines) in 2006–2007 (data as used in Fig. 4).

To explore the effects of environment in more detail, the data from the transgenic experiment and the HEALTHGRAIN multisite dataset (as used for Fig. 4) were combined (Fig. 7). It is notable that the samples from the transgenic experiment (shown as orange points and labeled RRes‐2013‐UK in Fig. 7) fall within the middle of the plot, with the nontransgenic genotypes grown in the United Kingdom in 2006–2007 (shown as pale blue points in Fig. 7) being most clearly separated from the other sites. This may relate to the fact that 2007 was a very cool wet year in the United Kingdom, as discussed by 12.

## 4 Concluding remarks

Metabolomic analysis is widely used in plant science, including cereal research 5. We have used 1H‐NMR spectroscopy of unfractionated polar extracts of milled wheat grain as a rapid high‐throughput method to determine diversity within and between species, and to quantify individual components which may affect the quality of wheat‐based foods for human consumption (choline, glycine betaine, and asparagine). It also allows the effects of various factors on the wider metabolome and on the concentrations of individual components to be monitored, e.g. the effects of environmental conditions or crop husbandry and nutrition. The effects of processing on grain components and metabolites generated by microbiological fermentation can also be determined 34. Finally, we have shown that 1H‐NMR spectroscopy can be used to monitor unintended effects on grain composition resulting from transgenic manipulation, with the robustness of the system allowing comparisons between datasets obtained on different occasions.

However, it should also be noted that many phytochemicals, such as phenolics, are not readily determined by NMR and in such cases either MS‐based methods 5, 31, 35 or other approaches may be required.

*The authors have declared no conflict of interest*.
